# A novel non-invasive sweat lactate biosensor and its application in human exercise monitoring

**DOI:** 10.3389/fbioe.2025.1661224

**Published:** 2025-09-16

**Authors:** Haolin Xin, Zilin Wei, Yingkai Qin, Aili Wei, Kang Chen, Longfei Xu, Bin Li, Kun Wang, Tianhui Wang

**Affiliations:** ^1^ Military Medical Sciences Academy, Tianjin, China; ^2^ Tianjin Key Laboratory of Exercise Physiology & Sports Medicine, Tianjin University of Sport, Tianjin, China; ^3^ No. 950 Hospital of the Chinese People’s Liberation Army, Yecheng, China

**Keywords:** sweat lactate, non-invasive biosensor, upconversion nanoparticles, MoS_2_ nanosheet, aptamer

## Abstract

**Introduction:**

Lactate is a key biomarker for clinical diagnostics and athletic performance monitoring. Conventional blood-based assays are invasive and not ideal for real-time applications. Sweat, as a non-invasive alternative, offers significant advantages for dynamic lactate tracking.

**Methods:**

We developed a highly sensitive fluorescence resonance energy transfer (FRET)-based aptasensor for lactate detection in sweat. The sensing platform utilizes aptamer-functionalized core–shell upconversion nanoparticles (APT-CS-UCNPs) as energy donors and Fe_3_O_4_-decorated molybdenum disulfide (MoS_2_) nanosheets as quenchers. In the absence of lactate, efficient FRET occurs due to the close proximity (<10 nm) between the donor and acceptor, quenching fluorescence at 545 nm. Lactate binding induces conformational changes in the aptamer, increasing donor–acceptor distance and restoring fluorescence intensity.

**Results:**

The aptasensor exhibited a broad linear detection range (0–30 mM, *R* ^2^ = 0.9981) and an ultralow detection limit (0.07785 mM), outperforming most reported electrochemical sensors. In spiked sweat samples, recovery rates ranged from 98.45% to 104.28%, with negligible cross-reactivity to common interferents. Comparative analysis with commercial kits and previously published methods confirmed superior sensitivity and ease of operation.

**Discussion:**

This FRET-based aptasensor enables accurate, rapid, and non-invasive lactate quantification using standard laboratory instrumentation. Its successful application in real human sweat samples highlights strong potential for both clinical diagnostics and athletic performance monitoring.

## 1 Introduction

Lactate, a critical metabolite in carbohydrate and non-essential amino acid pathways, is generated during anaerobic metabolism and exists in two chiral forms: L-lactate and D-lactate (Hofvendahl and Hahn-Hägerdal, 2000; Xiao et al., 2022; Khrais et al., 2022). Of these, L-lactate is the predominant isomer in human physiology and serves as a vital biomarker for clinical diagnostics, disease management, and athletic performance optimization (Ma et al., 2022; Schmiedeknecht et al., 2022; He et al., 2024; Ansari et al., 2025). Traditional lactate detection relies on invasive blood sampling, which poses risks such as discomfort and infection. In contrast, sweat-based monitoring offers a non-invasive alternative, as sweat contains lactate at concentrations of 5–25 mM and reflects real-time physiological states (Luo et al., 2021; Nunes et al., 2021; Brunmair et al., 2021; Jiang et al., 2022; Villiger et al., 2018; Xuan et al., 2021). This approach improves patient compliance, minimizes infection risks, and enables continuous health monitoring.

Conventional analytical methods like gas chromatography and high-performance liquid chromatography deliver high accuracy but are impractical for routine use due to their cost, complexity, and lack of portability (Onor et al., 2017; Calderón-Santiago et al., 2014; Bollella et al., 2019; Chen et al., 2024; Yu et al., 2024; Wen et al., 2024; Wei et al., 2023; Qu et al., 2023; Liu et al., 2025). Fluorescence-based biosensors address these limitations by offering rapid, sensitive, and user-friendly detection. Fluorescence resonance energy transfer systems, in particular, excel due to their ability to transfer energy efficiently between paired molecules (donors and acceptors) (Melnychuk et al., 2022; Algar et al., 2019; Geldert et al., 2017). When coupled with aptamers-synthetic molecules with high target specificity-FRET-based sensors achieve exceptional precision (Kang et al., 2020; A et al., 2020; Qi et al., 2022; Chen et al., 2022; Wang et al., 2009). For example, aptamer-FRET systems have been successfully applied to detect contaminants like kanamycin in food (Zhang et al., 2021). However, conventional fluorescent labels often suffer from instability, toxicity, and reliance on ultraviolet light, hindering their use in biological environments.

To address these challenges, rare-earth-doped upconversion nanoparticles (UCNPs) have emerged as a promising tool. UCNPs convert near-infrared light into visible emissions, minimizing background noise and enabling sensitive detection in complex samples (Francois, 2004; Wang et al., 2017; Arai and Camargo, 2021; Yao et al., 2019; Mendez-Gonzalez et al., 2018; Tsang et al., 2015; Wang et al., 2024). Advances in core-shell UCNP (CS-UCNP) design have further improved sensitivity (Guo et al., 2021; Luo et al., 2018; Liu et al., 2021). Simultaneously, MoS_2_ has attracted significant interest in biosensing owing to its unique optical and structural properties, including strong light absorption, high charge carrier mobility for efficient signal generation, and a defect-rich surface that promotes biomolecule adhesion (Wang et al., 2024; Ying et al., 2015; Srinivasan et al., 2016; Lembke et al., 2015). To enhance its utility in sensing applications, we incorporated *in-situ* synthesized Fe_3_O_4_ nanoparticles into the MoS_2_ matrix (Yu et al., 2015; Wei et al., 2021; Zhu et al., 2020). This integration introduces magnetic functionality, enabling rapid separation of target-bound complexes from biological samples through an external magnetic field. Such magnetic separation effectively reduces nonspecific background interference, thereby improving both the sensitivity and reliability of fluorescence-based detection (Ying et al., 2015; Ma et al., 2023; Xiao et al., 2024). Together, these innovations provide a robust framework for next-generation biosensors.

In this work, we introduce a novel lactate detection platform combining L-lactate-specific aptamers, CS-UCNPs, and magnetic MoS_2_(Fe_3_O_4_-MoS_2_) to achieve high sensitivity. The aptamers are immobilized on CS-UCNPs, forming fluorescent probes that selectively bind lactate. Magnetic MoS_2_, engineered for strong fluorescence quenching and aptamer adsorption, serves as the energy acceptor. When lactate binds to the aptamer, the resulting structural change weakens its interaction with MoS_2_. After magnetic separation, the fluorescence intensity at 545 nm is measured, with signal strength directly proportional to lactate concentration. The *in-situ* synthesis of Fe_3_O_4_ nanoparticles on MoS_2_ nanosheets imparts superparamagnetism to the quencher. This enables rapid (<1 min) magnetic separation of the Fe_3_O_4_-MoS_2_/aptamer complex from the solution phase after lactate binding. Crucially, it isolates unbound aptamer-UCNPs probes in the supernatant, minimizing nonspecific background interference and amplifying the fluorescence signal recovery. The magnetic functionality eliminates centrifugation/washing steps, streamlining detection and enhancing reproducibility. The integration of Fe_3_O_4_ introduces magnetic functionality, enabling rapid separation of target-bound complexes via an external magnetic field. This reduces nonspecific background interference, enhancing sensitivity and reliability. This relationship enables precise quantitative analysis, offering a reliable and non-invasive method for continuous physiological monitoring.

## 2 Materials and methods

### 2.1 Materials

Polyethyleneimine (PEI), glutaraldehyde (C_5_H_8_O_2_), ferrous sulfate heptahydrate (FeSO_4_-7H_2_O), sodium acetate (CH_3_COONa), ammonium fluoride (NH_4_F), dopamine hydrochloride (C_8_H_11_NO_2_·HCl), sodium hydroxide (NaOH), methanol (CH_3_OH), ammonia water (NH_3_·H_2_O), sodium molybdate (Na_2_MoO_4_·2H_2_O), glucose (C_6_H_12_O_6_), pyruvic acid (C_3_H_4_O_3_), citric acid (C_6_H_8_O_7_), 3-hydroxybutyric acid (C_4_H_8_O_3_), acetic acid (C_2_H_4_O_2_), L-alanine (C_3_H_7_NO_2_), L-cysteine (C_3_H_7_NO_2_S), glycine (C_2_H_5_NO_2_), and L-serine (C_3_H_7_NO_3_) were sourced from Macklin Biochemical Technology Co., Ltd. Yttrium chloride hexahydrate (YCl_3_·6H_2_O), ytterbium chloride hexahydrate (YbCl_3_·6H_2_O),Erbium chloride hexahydrate (ErCl_3_·6H_2_O), oleic acid (C_18_H_34_O_2_), 1-octadecene (C_18_H_36_), Tris-HCl buffer, and erbium chloride hexahydrate (ErCl_3_·6H_2_O) were obtained from Sigma-Aldrich Co., United States of America. Ferric chloride hexahydrate (FeCl_3_·6H_2_O), thiourea ((NH_2_)_2_CS), citric acid monohydrate (C_6_H_8_O_7_·H_2_O), and PEG-20000 (HO(C_2_H_4_O)_n_H) were procured from Shanghai Yi En Chemical Technology Co. SA. Lactate standard (C_3_H_6_O_3_) was acquired from Shanghai Yuan Ye Biotechnology Co., and cyclohexane (C_6_H_12_) from Tianjin Damao Chemical Reagent Factory. L-lactate aptamer was synthesized by Shanghai Sangong Biotechnology Co., Ltd. and purified by high performance liquid chromatography. Sequence of L-lactic acid aptamer: 5‘-Biotin-TEG-GACGACGAGTAGCGCGTATGAATGCTTTTCTATGGAGTCGTC-3’ (Huang and Liu, 2023). The L - Lactate Detection Kit was sourced from Shanghai Beyotime Biotechnology Co., Ltd.

### 2.2 Equipment

The primary instruments and equipment utilized in the experiments include: A UV-visible spectrophotometer (TU-1901) from Beijing Pujinjie General Instrument Co. An XRD diffractometer (BrukerD8) produced by Bruker Dalton. A 980 nm semiconductor laser emitter (LOS-BLD-0980-2W) supplied by Hitech Photonics Co. A transmission electron microscope (TEM JEM-2100) from Nippon Electronics Co. An X-ray Photoelectron Spectroscopy Tester (ESCALAB-250Xi) and a Fourier Transform Infrared Spectrophotometer (Nicolet iN10) both from Thermo Fisher Scientific China Ltd. A fluorescence spectrophotometer (F97pro) from Shanghai Prism Technology Co. A zeta potential analyzer (Mastersizer-3000) from Malvern Panaco, United Kingdom.

### 2.3 Preparation of CS-UCNPs

According to the solvothermal method for synthesizing UCNPs, 0.8 mmol of YCl_3_·6H_2_O, 0.18 mmol of YbCl_3_·6H_2_O, and 0.02 mmol of ErCl_3_·6H_2_O, along with 9 mL of OA and 15 mL of 1-ODE, were added into a 100 mL three-necked flask and fully dissolved to form a solution. The mixture was heated to 120 °C to facilitate vacuum degassing and dehydration. After the mixture cooled to room temperature, 10 mL of a methanol solution containing 4 mmol of ammonium fluoride (NH_4_F) and 2.5 mmol of sodium hydroxide (NaOH) was gradually added. The temperature was then adjusted to 20 °C and the solution was stirred for 30 min. Subsequently, the setup was switched to nitrogen purge mode and the temperature was raised to 120 °C to evaporate the methanol via condensation and reflux. Under a nitrogen atmosphere, the temperature of the system was increased to 320 °C and maintained for 1.5 h. Once the reaction concluded, the mixture was allowed to cool to room temperature, and ethanol was added to precipitate the nanoparticles. The resulting precipitate was centrifuged at 13,000 rpm for 15 min, washed thrice with cyclohexane, and then placed in a drying oven where it was vacuum-dried at 60 °C overnight. Finally, the dried precipitate was dispersed in cyclohexane for further use.

To encapsulate the NaYF_4_ shell onto the mononuclear upconversion nanoparticles using the layer-by-layer assembly method, 1 mmol of YCl_3_·6H_2_O was dissolved in 9 mL of OA and 15 mL of 1-ODE in a 100 mL three-necked flask. The mixture was heated to 120 °C under a nitrogen atmosphere for degassing and underwent vacuum dehydration. Upon reaching room temperature, the cyclohexane-dispersed mononuclear UCNPs, prepared previously, were incrementally introduced to the flask. Following this, 10 mL of a methanol solution containing 4 mmol of NH_4_F and 2.5 mmol of NaOH was added dropwise. The mixture was then stirred at room temperature for 30 min. The temperature was subsequently raised to 120 °C to enable condensation and reflux, facilitating the removal of methanol and cyclohexane. The system was further heated to 320 °C under a nitrogen atmosphere and maintained at this temperature for 1.5 h. Post-reaction, the product was washed three times with ethanol and cyclohexane before being stored.

### 2.4 Preparation of magnetic MoS_2_

Dissolve 1.21 g of Na_2_MoO_4_·2H_2_O, 1.52 g of (NH_2_)_2_CS, and 30 mg of PEG-20000 in 30 mL of deionized water, stirring vigorously at room temperature for 30 min. Transfer the mixture to a Teflon-lined autoclave and place it in a muffle furnace, heating to 220 °C for 24 h. After the reaction, the solid is washed four times with deionized water and subjected to vacuum freeze-drying to obtain solid MoS_2_. To prepare MoS_2_ nanosheets, disperse 100 mg of the solid MoS_2_ in 75 mL of Tris-HCl buffer (0.01 M, pH 8.5) and sonicate for 2 h.

Add 150 mg of C_8_H_11_NO_2_·HCl to the prepared solution and oscillate at room temperature for 4 h. Wash the resulting product three times with ultrapure water and subject it to vacuum freeze-drying to obtain polydopamine-functionalized MoS_2_ nanosheets. Subsequently, dissolve 100 mg of the functionalized material in 200 mL of water and sonicate for 5 min. Then, fully dissolve 6.0 g of FeCl_3_·6H_2_O and 3.1 g of FeSO_4_·7H_2_O in the solution and oscillate vigorously for 1 h. Quickly add 20 mL of 25% ammonia water and 7 g of citric acid to the mixture, and continue to oscillate vigorously for another hour. Filter and wash the resultant black precipitate six times with acetone and dry at 65 °C for 1 h to obtain the final product.

### 2.5 Probe preparation

Surface modification of CS-UCNPs was performed using a ligand exchange method, detailed as follows: Initially, 300 mg of PEI was fully dissolved in 5 mL of pure water in a beaker. Then, 10 mL of CS-UCNPs solution (2 mg/mL in cyclohexane) was added, and the mixture was stirred at room temperature for 24 h. After the reaction, the mixture was centrifuged at 12,000 rpm for 15 min to collect the precipitate, which was then washed multiple times with ethanol. Following a 6-h freeze-drying process, PEI-coated CS-UCNPs (PEI-CS-UCNPs) were obtained. Subsequently, sialic acid (SA) was attached to the surface of PEI-CS-UCNPs using the classic glutaraldehyde coupling method. The process involved uniformly mixing 1 mL of Tris-HCl buffer (0.01 M, pH 7.0) with 2 mg of PEI-CS-UCNPs and 250 μL of a 25% glutaraldehyde solution, followed by sonication for 10 min. The mixture was then gently oscillated at room temperature for 2 h. After centrifugation and washing, the precipitate was resuspended in Tris-HCl solution, to which 100 μL of 2 mg/mL SA was added. The mixture was incubated with oscillation at room temperature overnight to yield core-shell UCNPs coupled with SA (SA-CS-UCNPs). Finally, the prepared SA-CS-UCNPs (1 mL, 2 mg/mL) were incubated with biotin-modified lactate aptamers (50 μL, 10 μM) at 37 °C with slow oscillation overnight. Following incubation, the mixture was centrifuged, washed three times with Tris-HCl buffer, and resuspended for storage, resulting in the preparation of Apt-CS-UCNPs.

### 2.6 Optimisation of experimental parameters

Optimize the detection conditions for lactate using Apt-CS-UCNPs, several parameters were adjusted as follows. Three parallel groups were set up for each experiment.

Optimization of Buffer pH: 70 μL of Apt-CS-UCNPs (2 mg/mL) dispersed in Tris-HCl buffer was transferred to a centrifuge tube. The pH of the solution was adjusted to various values (6.5, 7.0, 7.4, 8.0, 8.8), and the volume was brought to 100 μL. After adding 100 μL of lactate, the mixture was incubated at 37 °C for 30 min. Subsequently, 100 μL of magnetic Fe_3_O_4_-MoS_2_ solution (1 mg/mL in Tris-HCl buffer) was added, thoroughly mixed, and the reaction continued at 37 °C for another hour.

Optimization of Fe3O4-MoS2 Concentration: 100 μL of Apt-CS-UCNPs (2 mg/mL) in Tris-HCl buffer (pH 7.0) was added to a centrifuge tube, followed by 100 μL of lactate standard. The mixture was incubated at 37 °C for 30 min. Different concentrations of magnetic Fe_3_O_4_-MoS_2_ solution (0, 0.2, 0.4, 0.6, 0.8 mg/mL in Tris-HCl buffer) were then added (100 μL each), mixed thoroughly, and incubated at 37 °C for 1 h.

Optimization of Reaction Time Between Fe_3_O_4_-MoS_2_ and UCNPs Probe: Starting with 100 μL of Apt-CS-UCNPs (2 mg/mL) in Tris-HCl buffer (pH 7.0) and 100 μL of lactate standard, the mixture was incubated at 37 °C for 25 min. Then, 100 μL of magnetic Fe_3_O_4_-MoS_2_ solution (0.4 mg/mL in Tris-HCl buffer) was added and the reaction was conducted at 37 °C for various times (0, 10, 20, 30, 40 min).

Optimization of Reaction Time Between UCNPs Probe and Target: 100 μL of Apt-CS-UCNPs (2 mg/mL) in Tris-HCl buffer (pH 7.0) was combined with 100 μL of lactate standard and incubated at 37°C for varying durations (0, 10, 20, 30, 40 min). This was followed by the addition of 100 μL of magnetic Fe_3_O_4_-MoS_2_ solution (0.4 mg/mL in Tris-HCl buffer), mixed well, and reacted at 37°C for 1 h.

After each optimization step, the centrifuge tube was placed on a magnetic rack for 1 min to facilitate magnetic separation. The supernatant was then transferred to a fluorescence cuvette, and the fluorescence intensity was measured at 545 nm under excitation at 980 nm using a fluorescence spectrophotometer. To ensure the accuracy of the results, each sample was tested in triplicate as part of parallel control experiments.

### 2.7 Detection of lactate

A total of 100 µL of Apt-CS-UCNPs (2 mg/mL) dispersed in Tris-HCl buffer (pH 7.0) was transferred into a centrifuge tube. Subsequently, 100 µL of lactate standard at varying concentrations (0–30 mM) was added, and the mixture was incubated at 37 °C for 20 min. Following this, 100 µL of magnetic Fe_3_O_4_-MoS_2_ solution (0.4 mg/mL) was introduced into the tube. After completion of the reaction, the tube was placed on a magnetic rack to facilitate magnetic separation for 1 min. The supernatant was then transferred to a fluorescence cuvette, and the fluorescence intensity at 545 nm was measured using a fluorescence spectrophotometer with excitation at 980 nm. To ensure the accuracy of the experimental results, each test sample was evaluated in three sets of parallel control experiments. Three parallel groups were set up for each experiment.

### 2.8 Using a fluorescent probe for specific lactate detection

To assess the specific selectivity of the fluorescent probe, several common potential interfering analytes were selected for evaluation, including glucose, pyruvic acid, citric acid, 3-hydroxybutyric acid, acetic acid, L-alanine, L-cysteine, glycine, and L-serine.

A lactate standard solution with a concentration of 5 mM was prepared using a Tris-HCl buffer solution. At the same time, solutions of interfering substances such as glucose and pyruvate were respectively prepared using the same Tris-HCl buffer solution, and their concentrations were all set to be 3 times the concentration of lactate (i.e., 15 mM) to evaluate the anti-interference ability of the detection system. The detection process strictly followed the steps described in Section 2.7 of this paper. 100 μL of the above-prepared solutions were sequentially taken and added to the detection system. By measuring the fluorescence intensity changes of the Apt-CS-UCNPs/Fe_3_O_4_-MoS_2_ fluorescent probe, the quantitative analysis of the target substance concentration was achieved. Three parallel groups were set up for each experiment.

### 2.9 Human sweat lactate test

In accordance with the criteria of having no organic diseases in the body, normal cardiopulmonary function, no skin diseases, no symptoms of exercise contraindications, and being able to complete a 40-min moderate-intensity power bike ride, 8 male subjects aged 24–28 years old, with a height of 175 ± 4 cm and a weight of 68 ± 5 kg were recruited before the experiment. Before the experiment, the experimental requirements and potential risks were explained in detail to them, and they signed a written informed consent form. The subjects were also required to avoid high-intensity exercise 2 days before the experiment, maintain a regular diet, refrain from smoking, drinking alcohol, and consuming foods containing caffeine. They were asked to clean their faces and heads 3 h before the experiment, and enter the experimental site with a room temperature of 26 °C and a relative humidity of 40% 30 min before the experiment. They changed into unified clothing and cleaned the sweat collection area on their foreheads. Subsequently, in a constant temperature and humidity environment, the subjects first rode at an intensity of 50 W for 3 min as a warm-up, and then rode at a rhythm of increasing the power by 20 W every minute at 1-min intervals until the heart rate reached 85% of the maximum heart rate (HRmax). After maintaining this intensity for 10 min of riding, the power was gradually decreased within 5 min until the exercise stopped. Immediately after the exercise, the sweat on the subjects’ foreheads was collected into a 5 mL centrifuge tube using a sterile scraper. After the collected sweat was filtered and centrifuged, it was detected using the sweat lactate biosensor designed in this study. The specific measurement steps strictly followed those described in Section 2.7 of this paper. 100µL of the treated sweat was taken and added to the detection system. By measuring the fluorescence intensity changes of the Apt-CS-UCNPs/Fe_3_O_4_-MoS_2_ fluorescent probe, the quantitative analysis of the lactate concentration in the sweat was achieved.

## 3 Results and discussion

**FIGURE 1:**
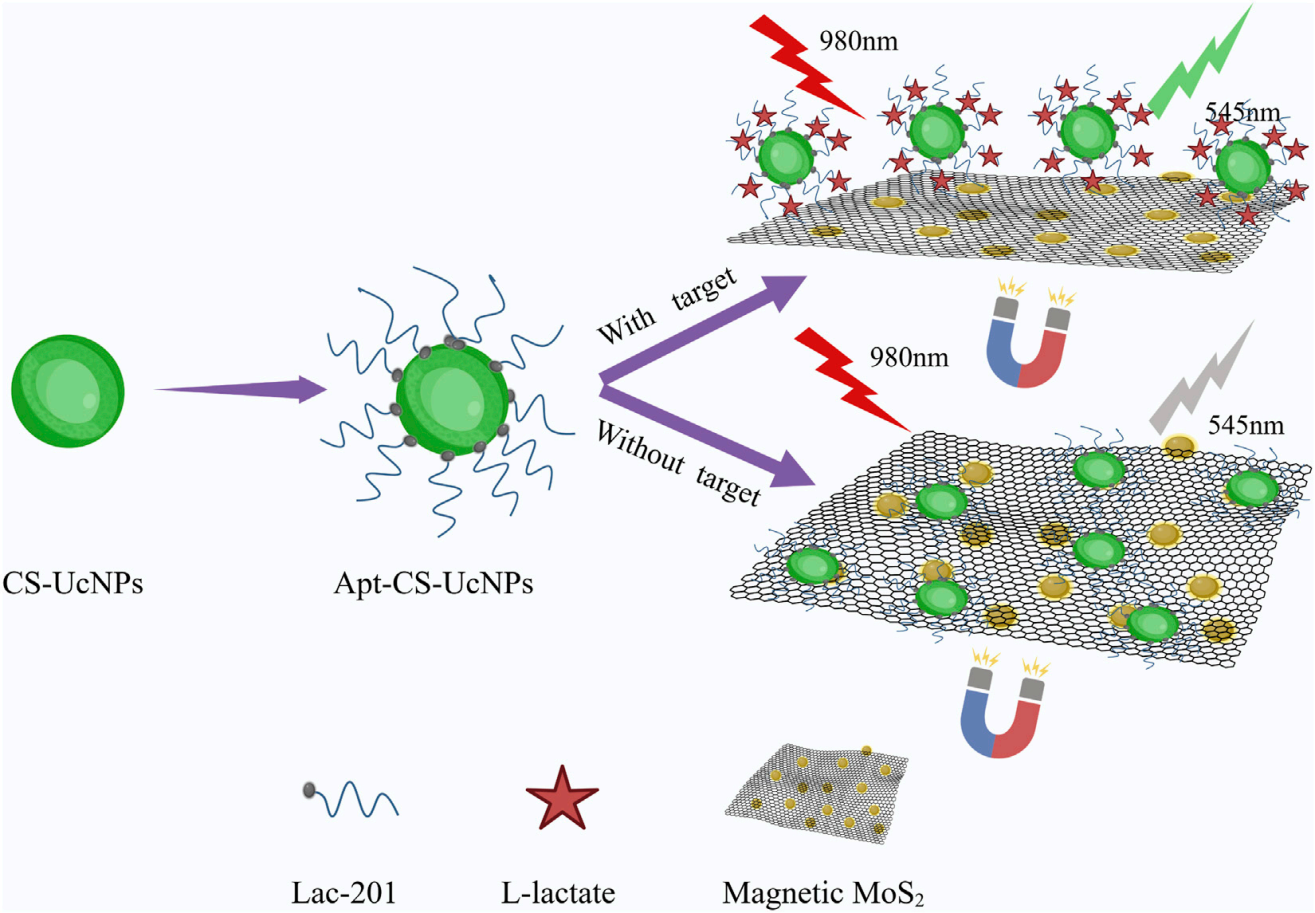
Schematic diagram of upconversion fluorescence assay for the detection of L-lactate in human sweat. The aptamers are immobilized on CS-UCNPs, forming fluorescent probes that selectively bind lactate. Magnetic MoS_2_, engineered for strong fluorescence quenching and aptamer adsorption, serves as the energy acceptor. When lactate binds to the aptamer, the resulting structural change weakens its interaction with MoS_2_. After magnetic separation, the fluorescence intensity at 545 nm is measured, with signal strength directly proportional to lactate concentration.

**FIGURE 2:**
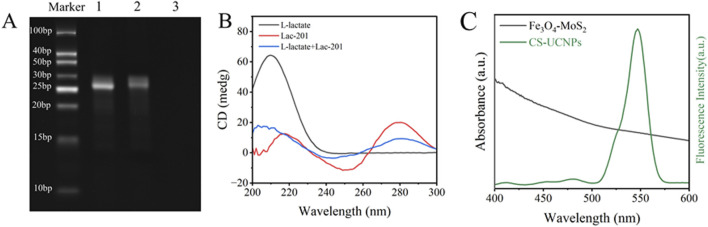
Feasibility Analysis of Lactate Biosensors: **(A)** Polyacrylamide gel electrophoresis (PAGE) with lane 1: Lac-201, lane 2: L-lactate + Lac-201, and lane 3: L-lactate; **(B)** Circular dichrogram of lactate and aptamer; **(C)** Fluorescence spectra of UCNPs and UV-absorption spectra of Fe_3_O_4_-MoS_2_.

The experimental design (Figure 1) aims to enhance fluorescence signals through the synthesis of core-shell upconversion nanoparticles (UCNPs: NaYF_4_:Yb,Er@NaYF_4_). Surface functionalization begins with ligand exchange to modify UCNPs with PEI, introducing amine groups for subsequent biomolecule conjugation. SA is covalently immobilized onto the aminated UCNPs using glutaraldehyde as a crosslinker. The biotinylated Lac-201 aptamer (Ansari et al., 2025; Huang and Liu, 2023), which specifically binds L-lactate, is then anchored to the SA-modified UCNPs via biotin-streptavidin interaction, forming a fluorescent energy donor probe. The structural specificity of the aptamer for L-lactate is confirmed by circular dichroism spectroscopy and non-denaturing polyacrylamide gel electrophoresis (Figures 2A,B). Concurrently, Fe_3_O_4_-MoS_2_ nanomaterials are synthesized as energy acceptors. These materials exhibit dual capabilities: strong fluorescence quenching of UCNPs in the UV region (Figure 2C) and high adsorption affinity for single-stranded DNA (ssDNA). The magnetic properties of MoS_2_ enable rapid separation of the nanomaterial from the solution phase. In the detection system, the UCNP-aptamer donor and MoS_2_ acceptor are combined. Upon L-lactate binding, the aptamer undergoes a conformational change from a flexible ssDNA state to a rigid target-bound structure, significantly reducing its adsorption to MoS_2_. Magnetic separation is then applied to remove MoS_2_ from the solution. Since unbound aptamers remain adsorbed onto MoS_2_, this step selectively retains the L-lactate-aptamer-UCNPs complexes in the supernatant. Fluorescence intensity at 545 nm is measured in the cleared supernatant, where the signal strength correlates linearly with L-lactate concentration, enabling quantitative detection via a pre-calibrated standard curve.

### 3.1 Characterisations of CS-UCNPs

**FIGURE 3:**
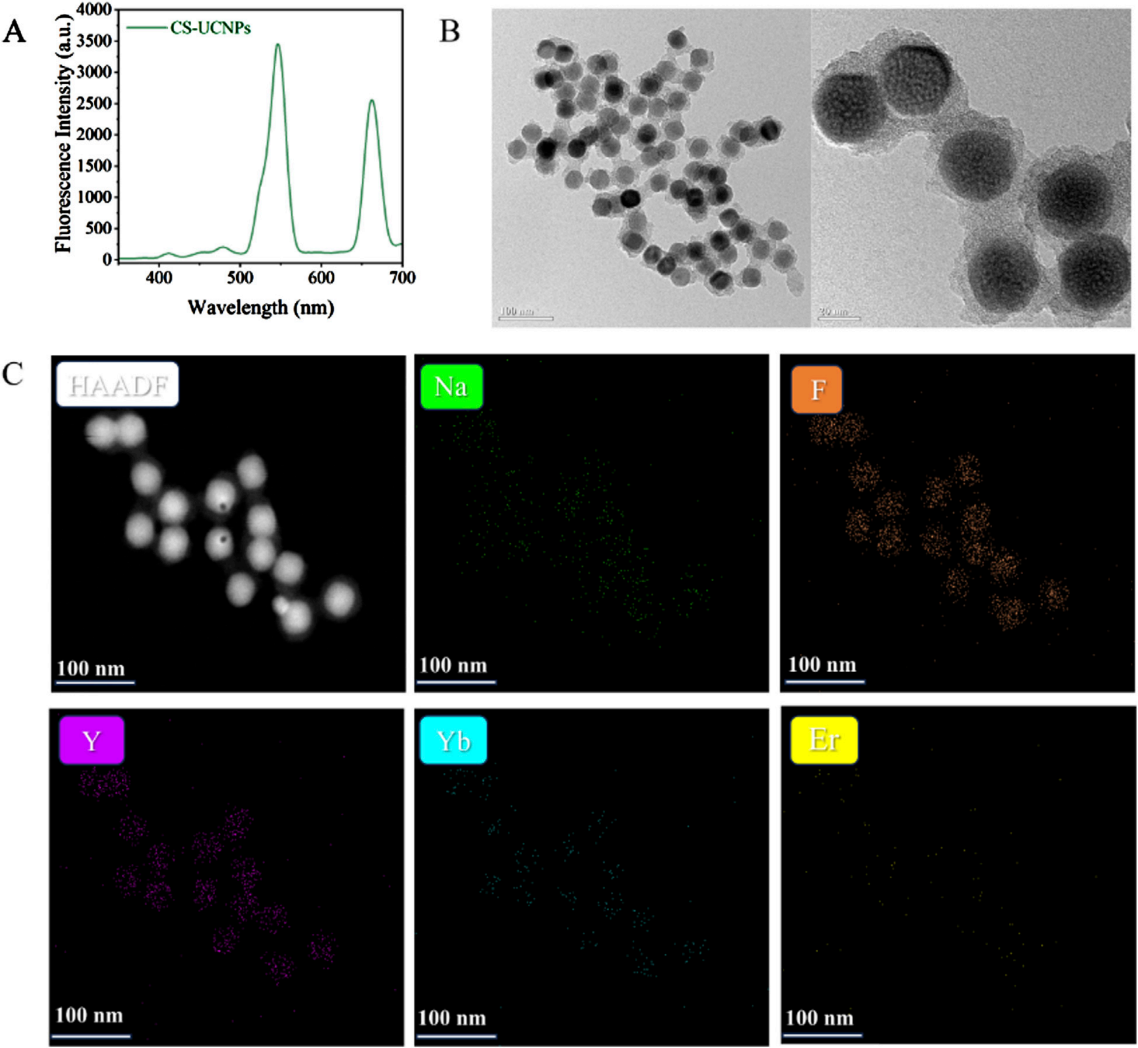
Characterisations of CS-UCNPs: **(A)** Fluorescence intensity of CS-UCNPs; **(B)** TEM and HRTEM of CS-UCNPs; **(C)** HAADF-STEM of CS-UCNPs.

The optical properties of the core-shell upconversion nanoparticles (CS-UCNPs) were first analyzed by fluorescence spectroscopy. A sharp emission peak at 545 nm was observed (Figure 3A), characteristic of the Er^3+^ ion transitions, demonstrating the successful energy upconversion capability of the nanoparticles. Complementing the optical analysis, transmission electron microscopy (TEM) revealed the morphological and structural attributes of the CS-UCNPs (Figure 3B), the nanoparticles exhibit a uniform spherical morphology with an average diameter of 28 nm and clear core-shell contrast, confirming the effective encapsulation of the NaYF_4_:Yb, Er core by the inert NaYF_4_ shell. The elemental composition and spatial homogeneity of the CS-UCNPs were further validated through high-angle annular dark-field scanning TEM(HAADF-STEM). Elemental mapping (Figure 3C) confirms the uniform distribution of yttrium (Y), fluorine (F), sodium (Na), ytterbium (Yb), and erbium (Er) across the nanoparticles, with distinct core-shell interfacial segregation.

### 3.2 Characterizations of Apt-CS-UCNPs

**FIGURE 4:**
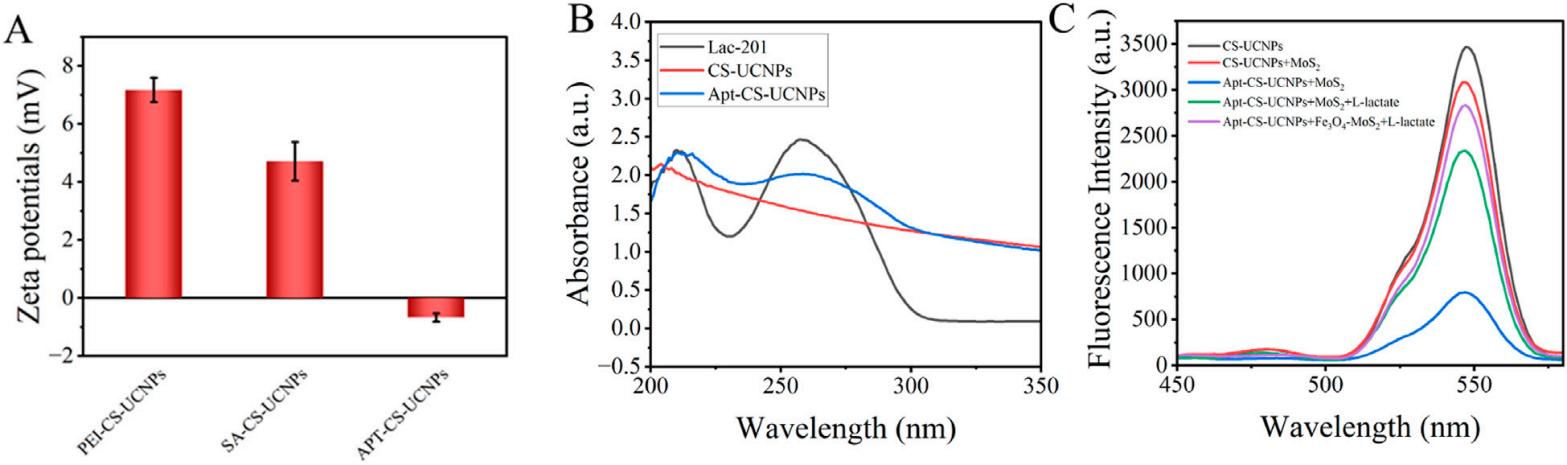
The combination of UCNPs and aptamers: **(A)** Zeta potentials of PEI-CS-UCNPs, SA-CS-UCNPs and APT-CS-UCNPs; **(B)** Changes in UV absorption spectra of UCNPs before and after binding aptamers. The black line is the spectrum of LAC 201 alone, the red line is the spectrum of UCNPs alone, and the blue line is the spectrum of LAC 201 combined with UCNPs; **(C)** Schematic diagram of verifying the affinity between Apt-CS-UCNPs and MoS_2_ using a fluorescence spectrophotometer.

The SA conjugation process on PEI-CS-UCNPs was monitored through zeta potential analysis (Figure 4A). The initial zeta potential of PEI-CS-UCNPs registered at +7.17 mV, consistent with the cationic nature of polyethyleneimine’s amine-rich surface. Upon SA crosslinking via glutaraldehyde, the zeta potential decreased to +4.71 mV, indicating partial neutralization of surface amines through covalent binding with SA. Subsequent aptamer immobilization further reduced the zeta potential to −0.67 mV, aligning with the negative charge of DNA (isoelectric point: 4–4.5) and confirming successful bioconjugation.

UV-Vis absorption spectroscopy provided complementary evidence for aptamer functionalization (Figure 4B). A distinct absorption peak emerged at 260 nm after aptamer conjugation-characteristic of DNA’s π-π* electronic transitions in nucleobases-which was absent in pristine CS-UCNPs. This optical signature, coupled with the systematic zeta potential shifts, conclusively demonstrates the stepwise assembly of the SA-aptamer complex on the nanoparticle surface. The reproducibility of these physicochemical changes underscores the robustness of the conjugation strategy for constructing target-specific fluorescent probes.

A fluorescence spectrophotometer was used to verify the affinity between Apt-CS-UCNPs and MoS_2_(Figure 4C). The experimental results showed that the CS-UCNPs system alone exhibited the highest fluorescence intensity. When MoS_2_ was added to the CS-UCNPs solution, the fluorescence intensity decreased significantly. It is speculated that this is due to the turbidity of the solution caused by the inherent properties of MoS_2_, which reduces the light transmittance. When Apt-CS-UCNPs were mixed with MoS_2_, the fluorescence intensity of the system dropped to the lowest level. This phenomenon indicates that MoS_2_ has a strong adsorption effect on the ssDNA modified on the surface of CS-UCNPs, which shortens the distance between CS-UCNPs, serving as an energy donor, and the acceptor MoS_2_ to within 10 nm, thus triggering an efficient FRET quenching effect. It is worth noting that when lactate was further added to the above system, the fluorescence intensity recovered significantly. This verifies that lactate specifically binds to the aptamer, causing a change in the spatial conformation of the aptamer, which leads to the distance between Apt-CS-UCNPs and MoS_2_ being greater than 10 nm, effectively blocking the FRET process. After endowing MoS_2_ with magnetic properties, the fluorescence intensity can be further restored under the effect of magnetic separation.

### 3.3 Characterisations of MoS_2_ and magnetic MoS_2_

**FIGURE 5:**
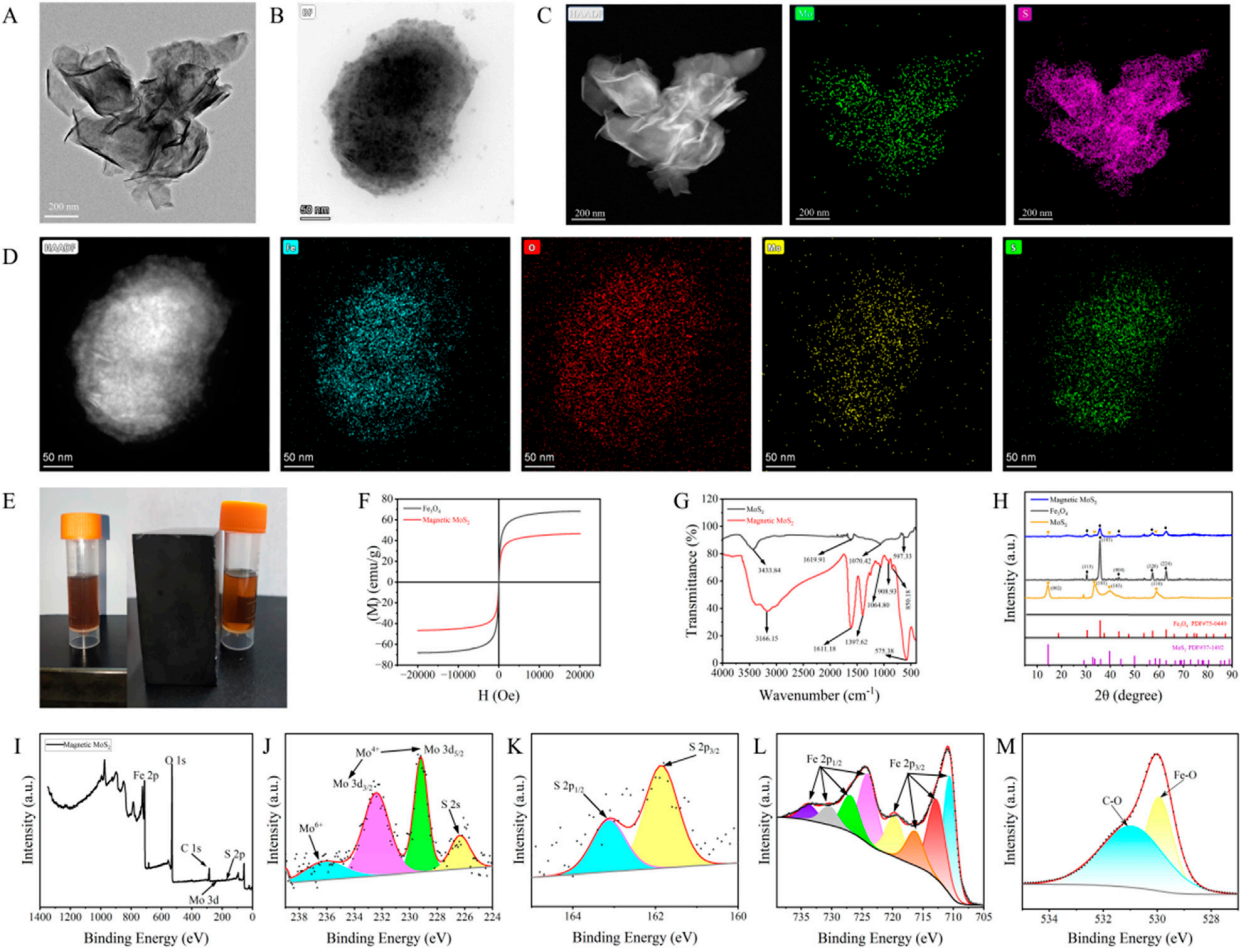
Synthesis and Characterizations of Magnetic MoS_2_: **(A)** TEM of MoS_2_; **(B)** TEM of magnetic MoS_2_; **(C)** HAADF-STEM of MoS_2_; **(D)** HAADF-STEM of magnetic MoS_2_; **(E)** The magnetic manifestations of MoS_2_ and magnetic MoS_2_ under the external magnetic environment; **(F)** The saturation magnetization of magnetic MoS_2_ (47 emu/g) and the superparamagnetism of Fe_3_O_4_ (70 emu/g); **(G)** The infrared spectrum of MoS_2_ and magnetic MoS_2_ (a new absorption peak at 575 cm^-1^ appeared, corresponding to the characteristic stretching vibration of the Fe-O bond in Fe_3_O_4_); **(H)** The X-ray diffraction (XRD) patterns of magnetic MoS_2_, MoS_2_, and Fe_3_O_4_(characteristic peaks belonging to both MoS_2_: 2θ = 14.59, 33.63, 39.44, 58.65° and Fe_3_O_4_: 2θ = 30.42, 35.89, 43.32, 57.05, 63.18°); **(I)** The X-ray photoelectron spectroscopy (XPS) survey spectrum of magnetic MoS_2_; **(J)** The binding energy peaks of tetravalent Mo at 229.17 eV and 232.49 eV can be attributed to Mo3d5/2 and Mo3d3/2, respectively; **(K)** The binding energy peaks of S at 161.86 eV and 163.14 eV can be attributed to S2p1/2 and S2p3/2; **(L)** The binding energy peaks of Fe at 724.03 eV, 726.91 eV, 730.41 eV, and 733.85 eV can be attributed to Fe2p1/2, and those at 710.59 eV, 712.79 eV, 716.31 eV, and 719.67 eV can be attributed to Fe2p3/2; **(M)** The binding energy peaks of O at 530.17 eV and 531.82 eV originate from Fe-O bonds and C-O bonds, respectively.

TEM imaging (Figure 5A) confirms the two-dimensional lamellar morphology of the synthesized MoS_2_ nanosheets, with lateral sizes ranging from 5 nm to 200 nm. Characteristic features such as stacked layers, edge wrinkling, and localized agglomeration are observed, consistent with typical MoS_2_ nanostructures. HAADF-STEM elemental mapping (Figure 5C) verifies the uniform spatial distribution of molybdenum (Mo) and sulfur (S) throughout the nanosheets. Quantitative analysis yields a Mo/S atomic ratio of 1:2, in exact agreement with the stoichiometric composition of MoS_2_. These results collectively confirm the successful synthesis of chemically homogeneous MoS_2_ nanosheets.

TEM imaging of the Fe_3_O_4_-MoS_2_ nanocomposite (Figure 5B) reveals MoS_2_ nanosheets (200–300 nm in lateral size) serving as substrates for the uniform deposition of Fe_3_O_4_ nanoparticles (5–10 nm). The nanoparticles exhibit dense, ordered alignment across the nanosheets, forming a hierarchical architecture with minimal aggregation. HAADF-STEM elemental mapping (Figure 5D) further demonstrates spatially correlated distributions of Fe, O, Mo, and S, confirming the successful integration of magnetic components with the MoS_2_ framework.

The Fe_3_O_4_-MoS_2_ nanocomposite demonstrates rapid magnetic separation capability, as evidenced by solution clarification within 1 min under an external magnetic field (Figure 5E). Vibrating sample magnetometry reveals a saturation magnetization of 47 emu/g (Figure 5F), approximately 67% of bare Fe_3_O_4_ nanoparticles (70 emu/g). FTIR analysis (Figure 7G) identifies critical bonding features: A broad band at 3,433 cm^-1^ corresponds to hydroxyl (-OH) stretching from surface-adsorbed water. The emergence of a distinctive peak at 575 cm^-1^, assigned to Fe-O stretching vibrations in Fe_3_O_4_, confirms covalent anchoring of nanoparticles onto MoS_2_. This confirms the successful loading of Fe_3_O_4_ nanoparticles onto the MoS_2_ surface and suggests that the interaction between the two components involves chemical bonding. The above results indicate that the quenching agent we synthesized has the dual functions of quenching and magnetic separation.

The crystalline phase evolution of the Fe_3_O_4_-MoS_2_ nanocomposite was investigated using X-ray diffraction (XRD) (Figure 5H). Pristine MoS_2_ exhibits characteristic peaks at 14.59° (002), 33.63° (101), 39.44° (103), and 58.65° (110), consistent with hexagonal-phase MoS_2_ (JCPDS 37–1492). Fe_3_O_4_ nanoparticles show diffraction peaks at 30.42° (220), 35.89° (311), 43.32° (400), 57.05° (511), and 63.18° (440), matching cubic spinel Fe_3_O_4_ (JCPDS 75–0449). Importantly, the composite retains all characteristic peaks of both components, confirming the *in-situ* growth of Fe_3_O_4_ on MoS_2_ without structural degradation.

Surface chemical states were further probed using X-ray photoelectron spectroscopy (XPS) (Figures 5I–M). The Mo 3d spectrum (Figure 5J) exhibits two dominant peaks at 229.17 eV (Mo^3+^ 3d_5_/2) and 232.49 eV (Mo^3+^ 3d3/2), unambiguously confirming the MoS_2_ phase, while a weak satellite peak at 236.16 eV suggests partial surface oxidation to Mo^6+^ during synthesis. The S 2p spectrum (Figure 5K) displays characteristic doublet peaks at 161.86 eV (S^2-^ 2p3/2) and 163.14 eV (S^2-^ 2p_1_/2), consistent with S-Mo covalent bonding in MoS_2_. In the Fe 2p region (Figure 5L), the 2p3/2 orbital manifests four distinct peaks at 710.59 eV (Fe^2+^), 712.79 eV (Fe^3+^), 716.31 eV, and 719.67 eV, while the 2p_1_/2 orbital shows corresponding peaks at 724.03 eV, 726.91 eV, 730.41 eV, and 733.85 eV. This multi-peak configuration, characteristic of mixed Fe^2+^/Fe^3+^ oxidation states, provides definitive evidence for the magnetite (Fe_3_O_4_) phase. Additionally, the O 1s spectrum (Figure 5M) reveals a dominant Fe-O bond contribution at 530.17 eV and a minor component at 531.82 eV attributed to surface hydroxyl groups, collectively verifying the chemical integrity of the Fe_3_O_4_-MoS_2_ heterostructure. The *in-situ* synthesis of Fe_3_O_4_ nanoparticles on MoS_2_ nanosheets imparts superparamagnetism to the quencher. This enables rapid (<1 min) magnetic separation of the Fe_3_O_4_-MoS_2_/aptamer complex from the solution phase after lactate binding. Crucially, it isolates unbound aptamer-UCNPs probes in the supernatant, minimizing nonspecific background interference and amplifying the fluorescence signal recovery. The magnetic functionality eliminates centrifugation/washing steps, streamlining detection and enhancing reproducibility. The integration of Fe_3_O_4_ introduces magnetic functionality, enabling rapid separation of target-bound complexes via an external magnetic field. This reduces nonspecific background interference, enhancing sensitivity and reliability.

### 3.4 Optimization of experimental conditions

**FIGURE 6:**
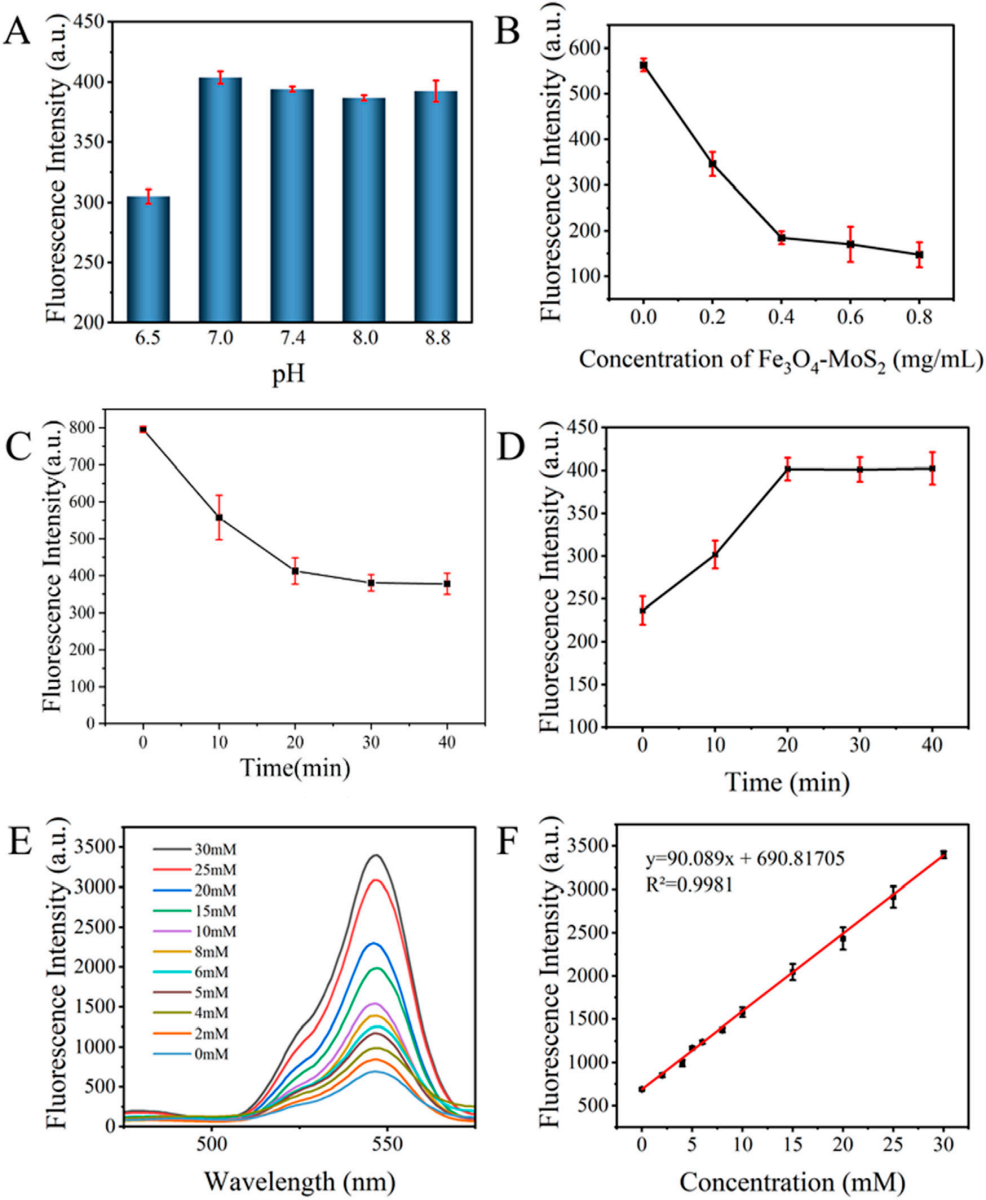
Optimization and Linear Analysis of the Sweat Lactate Biosensor: **(A)** pH; **(B)** Concentrations of Fe_3_O_4_-MoS_2_; **(C)** Reaction time between Apt-CS-UCNPs and Fe_3_O_4_-MoS_2_; **(D)** Reaction time between aptamer and lactate on lactate detection; **(E)** Fluorescence spectra of the detection system in the presence of different concentrations of lactate; **(F)** Linear relationship between different concentrations of lactate (0, 2, 4, 5, 6, 8 10, 15, 20, 25, 30 mM) and fluorescence intensity.

To enhance the accuracy and sensitivity of lactate detection, we systematically optimized key parameters governing the FRET-based biosensing system. Initially, we analyzed the effect of varying pH levels on the biosensor’s performance (Figure 6A). The highest fluorescence intensity occurred at pH 7, likely because this pH aligns with typical biological conditions, facilitating specific aptamer-lactate binding and stable FRET processes. Therefore, pH 7 was chosen as the optimal reaction condition.

Subsequently, analysis focused on Fe_3_O_4_-MoS_2_ concentration-dependent fluorescence quenching (Figure 6B). As Fe_3_O_4_-MoS_2_ concentration increased from 0 to 0.8 mg/mL, the fluorescence intensity progressively decreased due to enhanced FRET efficiency, reaching a plateau at 0.4 mg/mL where further concentration changes caused only marginal signal fluctuations, indicative of FRET interaction saturation between Apt-CS-UCNPs and Fe_3_O_4_-MoS_2_.

Additionally, investigation of incubation time effects (Figure 6C) revealed a time-dependent fluorescence decline that stabilized after 20 min, suggesting attainment of dynamic equilibrium between Apt-CS-UCNPs and Fe_3_O_4_-MoS_2_. This time point was selected to maximize both FRET efficiency and system stability.

Finally, critical validation was obtained from the lactate-triggered fluorescence recovery process (Figure 6D), where aptamers specifically bound to lactate molecules and underwent conformational changes, leading to their detachment from Fe_3_O_4_-MoS_2_ surfaces. This resulted in time-dependent restoration of quenched fluorescence–the intensity reached maximum recovery at 20 min and fluorescence stabilized, thereby confirming the aptamer-lactate binding kinetics and the dynamic response characteristics of the sensing system.

In summary, through systematic optimization of key parameters, we identified ideal experimental conditions: 20 min of incubation for both Apt-CS-UCNPs with Fe_3_O_4_-MoS_2_ and the sensing system with lactate, conducted at 37°C and pH 7.0. These optimized conditions provide a solid foundation for precise and sensitive lactate detection.

### 3.5 Design of fluorescent probes and mechanism of lactate detection

We constructed a FRET biosensor utilizing Apt-CS-UCNPs as energy donors and Fe_3_O_4_-MoS_2_ magnetic nanosheets as quenchers. The mechanism capitalizes on distance-modulated FRET behavior, where initial fluorescence quenching occurs through <10 nm donor-acceptor proximity. Target recognition induces aptamer-lactate binding that provokes conformational reorganization, displacing the CS-UCNPs beyond FRET-effective distances (>10 nm). This spatial reorganization terminates energy transfer, resulting in fluorescence recovery proportional to lactate concentration.

Under optimized detection conditions, the biosensor response was systematically assessed through incremental lactate additions (0–30 mM) with fluorescence monitoring at 545 nm (Figure 6E). As shown in Figure 6F, the system demonstrated proportional fluorescence recovery with increasing lactate concentrations, showing a linear correlation (*R* ^2^ = 0.9981) expressed as: y = 90.089x + 690.81705 where y represents fluorescence intensity (a.u.) and x denotes concentration (mM). The calculated detection limit of 0.07785 mM confirms high sensitivity across the tested range. LOD was determined via 3σ/k method: σ = SD of blank signals (n = 10) = 2.338 a. u., k = Slope of calibration curve = 90.089 a. u./mM, LOD = 3 × 2.338/90.089 = 0.07785 mM. This quantitative model enables precise lactate detection, supporting applications in biomedical research, environmental monitoring, and food safety analysis.

### 3.6 Selectivity of sweat lactate detection

**FIGURE 7:**
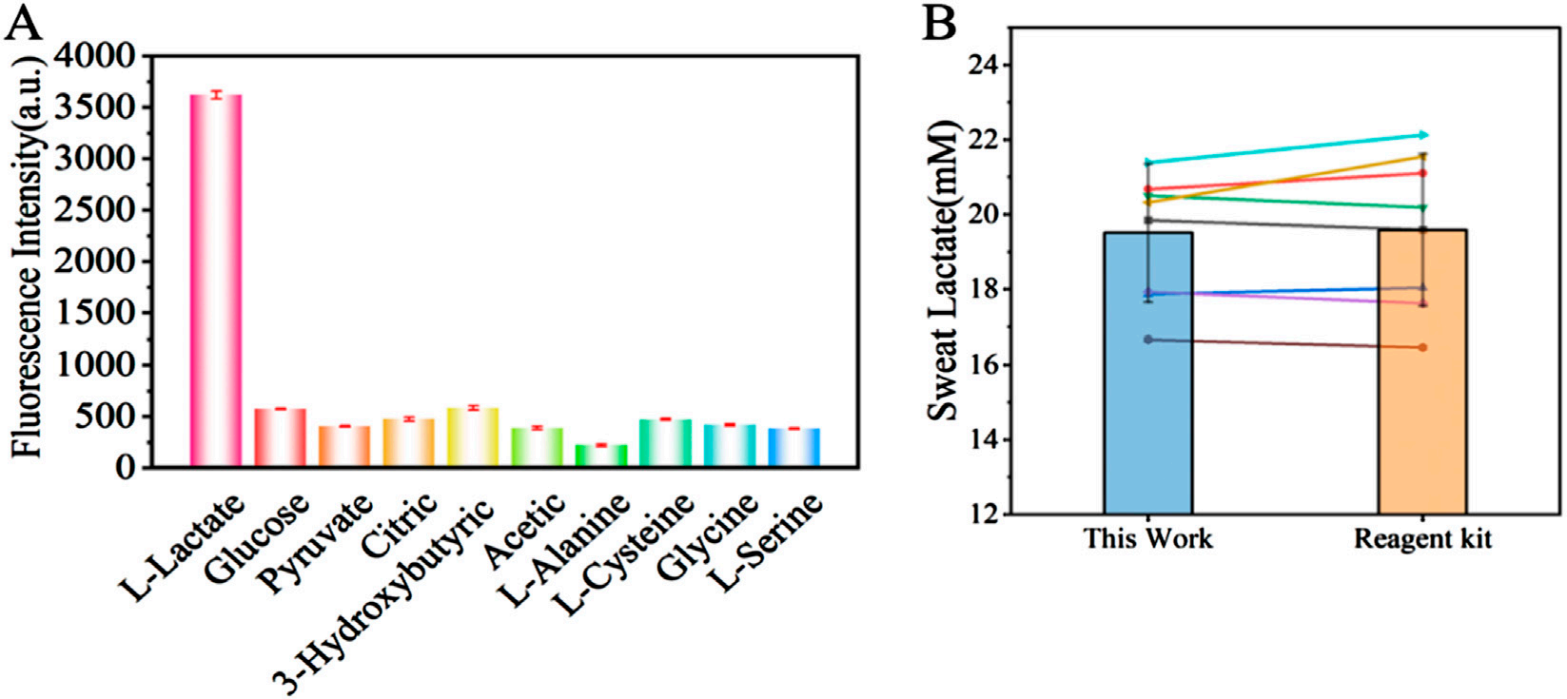
**(A)** Fluorescence intensity of the lactate biosensor during detection of interferents (Glucose, Pyruvate, Citric Acid, 3-Hydroxybutyric Acid, acetic acid, L-Alanine, L-Cysteine, Glycine, L-Serine). **(B)** Comparison of lactate concentration detection between the sweat lactate fluorescence biosensing technology developed in this study and a commercial lactate assay kit (n = 8).

To assess detection specificity, we evaluated nine physiological substances reported as potential interferents for L-lactate detection. Each compound-including glucose, pyruvate, citric acid, 3-hydroxybutyric acid, acetic acid, L-alanine, L-cysteine, glycine, and L-serine-was tested at concentrations threefold higher than the target L-lactate (30 mM) level. We used a mixture for specific analysis, and the explicit dosage of the additive is shown in Table 1. Notably, while glucose naturally exists at millimolar (mM) concentrations in blood, other interferents typically present at micromolar (μM) levels or lower, particularly in sweat. As shown in Figure 7A, exposure to interferent-only conditions caused significant fluorescence quenching at 545 nm. In contrast, L-lactate alone induced marked fluorescence enhancement. This stark response dichotomy conclusively demonstrates the method’s high specificity, attributable to the aptamer’s precise molecular recognition capability for L-lactate.

**TABLE 1:** Specificity experiment: the addition amounts of 10 groups of lactic acid interference mixtures.

Group	L-Lactate	Glucose	Pyruvate	Citric acid	3-Hydroxybutyric acid	Acetic acid	L-Alanine	L-Cysteine	Glycine	L-Serine
1	30 mM	90 mM	90 mM	90 mM	90 mM	90 mM	90 mM	90 mM	90 mM	90 mM
2	0	90 mM	30 mM	30 mM	30 mM	30 mM	30 mM	30 mM	30 mM	30 mM
3	0	30 mM	90 mM	30 mM	30 mM	30 mM	30 mM	30 mM	30 mM	30 mM
4	0	30 mM	30 mM	90 mM	30 mM	30 mM	30 mM	30 mM	30 mM	30 mM
5	0	30 mM	30 mM	30 mM	90 mM	30 mM	30 mM	30 mM	30 mM	30 mM
6	0	30 mM	30 mM	30 mM	30 mM	90 mM	30 mM	30 mM	30 mM	30 mM
7	0	30 mM	30 mM	30 mM	30 mM	30 mM	90 mM	30 mM	30 mM	30 mM
8	0	30 mM	30 mM	30 mM	30 mM	30 mM	30 mM	90 mM	30 mM	30 mM
9	0	30 mM	30 mM	30 mM	30 mM	30 mM	30 mM	30 mM	90 mM	30 mM
10	0	30 mM	30 mM	30 mM	30 mM	30 mM	30 mM	30 mM	30 mM	90 mM

### 3.7 Methodological comparison of lactate detection methods

To validate the biosensor’s practical accuracy, recovery tests were performed using a 2 mM L-lactate standard in synthetic sweat. Our system demonstrated recoveries of 98.45%–104.28%, exhibiting superior consistency compared to a commercial assay kit (97.59%–106.40%) under identical physiological conditions (Table 2). When benchmarked against reported methods (Table 3), the biosensor outperforms most optical/electrochemical sensors, achieving a broader linear detection range (0–30 mM) and a lower detection limit (0.07785 mM). The enzyme-free design not only eliminates reliance on lactate oxidase but also enhances operational stability and reduces costs. Combined with its accuracy, sensitivity, and environmental robustness, this biosensor establishes itself as a reliable platform for non-invasive sweat-based metabolic biomarker monitoring, with direct applications in sports physiology and early-stage disease diagnostics.

**TABLE 2:** Actual detection efficiency and Recover rate between the designed lactate biosensor and a kit for lactate detection.

Means	Brochure	Measured value (mM)	Recovery rate (%)
Reagent kit	L-Lactate Standard	2.1279	106.40
		1.9660	98.30
		1.9518	97.59
This work		2.086	104.28
		2.031	101.56
		1.969	98.45

**TABLE 3:** Comparison of the designed sweat lactate biosensor with other sweat lactate detection methods reported in literature.

Sensor type	Sensor Design	Linearity Ranges	Limit Of Detection	Response Time	Selectivity	Application Context	References
Flex-GO sensor	Graphene oxide (GO) nanosheets integrated into a nanoporous flexible electrode system	1-100 mM	1 mM	45 min	Cortisol	Synthetic sweat	Lin et al. (2020)
Non-enzymatic sensor	Based on screenprinted structures with the working surface modified in course of electropolymerization of 3-aminophenylboronic acid (3-APBA) with imprinting of lactate	3-100 mM	1.5 mM	2-3 min	/	Human sweat	Zaryanov et al. (2017)
MIP based screen printed carbon electrode (SPCE) sensors	Both cyclic voltammetry (CV) and differential pulse voltammetry (DPV) were used as modes of analysis for the MIP SPCE sensors	/	2.2 mM	About 1min	Uric acid, Ascorbic acid, Cortisol	Human sweat	Mugo et al. (2023)
Electrochemical Sensors	Nanocellulose derived from hemp (HNC) with the addition of silver nanoparticles (AgNPs) is utilized for improving the electrochemical sensing performances for lactate detection.	0-25 mM	0.56 mM	About 120 s	/	Human sweat	Phumma et al. (2024)
Electrochemical Biosensors	Blue (PB) nanoparticles as the internal redox probe on screen-printed carbon electrodes (SPCE), followed by a layer of electropolymerized MIP (eMIP) for molecular recognition, enabling reagent-free lactate detection	1-35 mM	0.20 mM	About 1200 s	Urea, Glucose, Acetaminophen, Ascorbic acid, Uric acid	Artificial sweat	Dykstra et al. (2024)
Visual ratiometric fluorescence sensing of L-lactate in sweat	Using 1, 4-H2NDC as ligand and Eu as the center metal, the lanthanide MOF Eu-NDC was synthesized by hydrothermal method. The material showed a fast ratiometric response to L-lactate, and the fluorescence of the material varied from red to blue with the growth of lactate concentration,	/	0.52 mM	5 min	Ca2+, Mg2+, Na+, Cl−, K+, NH4+, Urea	Artificial sweat	Jia and Yan (2023)
CS-UCNPs/Fe3O4-MoS2	Fluorescence resonance energy transfer (FRET) between CS-UCNPs and Fe3O4-MoS2	0-30 mM	0.07785 mM	About 45 min	Glucose, Pyruvate, Citric Acid, 3-Hydroxybutyric Acid, Acetic Acid, L-Alanine, L-Cysteine, Glycine, L-Serine	Human sweat	This work

Flex-GO, flexible graphene oxide; 3-APBA, 3-aminophenylboronic acid; MIPs, molecularly imprinted polymers; PB, prussian blue; SPCE, screen-printed carbon electrodes; HNC, nanocellulose derived from hemp; Ag NPs, silver nanoparticles; PVA, poly (vinyl alcohol); Lox, Lactate oxidase; NR, not reported.

### 3.8 Sweat lactate assay

**FIGURE 8:**
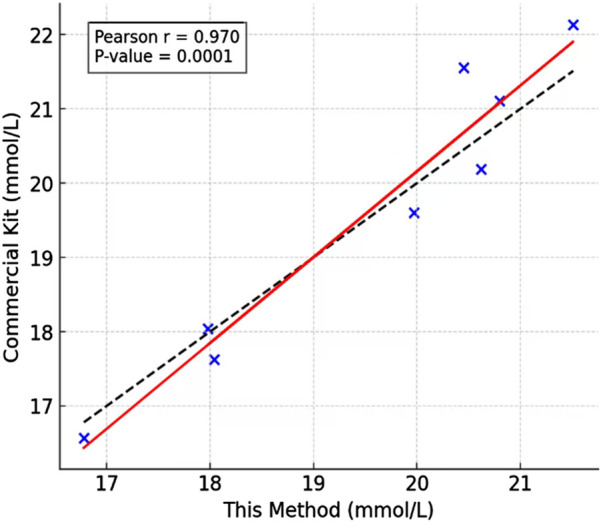
Sweat lactate assay comparison: Fitting regression line based on the least square method.

To validate the practical utility of our detection method, we analyzed sweat samples collected from human subjects through a standardized protocol, and compared the lactate concentrations in the sweat using the sweat lactate biosensing technology developed in this study and a commercial lactate kit. As shown in Figure 7B, there was no significant difference in the lactate concentrations in the sweat. The high linear correlation (r = 0.97) obtained by least squares regression analysis confirms that our method accurately tracks the concentration trends measured by the established standard, validating its reliability for quantitative analysis and confirming its effectiveness in the detection of real-world biological samples (as shown in Figure 8). Our assay design obviates the requirement for lactate oxidase, thereby substantially lowering the per-test expense. Secondly, the core sensing components—the synthetic fluorescent probe and the aptamer—exhibit markedly superior long-term stability and enhanced resistance to degradation compared to conventional enzyme-based reagents, resulting in an extended shelf life. Furthermore, the operational workflow is significantly streamlined, requiring only a single-step incubation and separation process leveraging recyclable Fe_3_O_4_-MoS_2_ nanocomposites for simultaneous target capture and signal amplification. This architecture not only augments detection sensitivity but also underscores the potential for sustainable, low-cost point-of-care diagnostic applications, as the magnetic nanocomposite can be efficiently retrieved and regenerated for repeated use.

This method’s non-invasive nature offers significant advantages over traditional invasive techniques. By enabling lactate measurement directly from sweat, it eliminates the discomfort and risks associated with blood sampling, improving both subject comfort and cooperation. The ability to non-invasively monitor lactate levels creates new opportunities for optimizing exercise programs and preventing overtraining syndromes. Coaches and healthcare providers can now obtain physiologically relevant data with minimal user burden, supporting evidence-based adjustments to training loads and recovery strategies.

## 4 Conclusion

This study successfully established a novel lactate biosensor based on a FRET system utilizing Apt-CS-UCNPs and Fe_3_O_4_-MoS_2_ nanosheets. The developed platform demonstrates outstanding selectivity and sensitivity, achieving precise quantitative detection of lactate within a linear range of 0–30 mM (*R* ^2^ = 0.9981) and a remarkably low limit of detection (LOD) of 0.07785 mM. Validation experiments confirmed high accuracy, with recovery rates of 98.45%–104.28% in complex matrices. Comparative analysis with existing methods and commercial kits highlighted the sensor’s superior sensitivity and exceptionally low LOD, while specificity tests verified its resistance to interference from common biomolecules. Magnetic separation selectively removes MoS_2_-bound complexes, isolating lactate-aptamer-UCNPs in the supernatant. This step is critical for noise reduction and signal amplification. Unlike non-magnetic MoS_2_, the Fe_3_O_4_-MoS_2_ nanocomposite uniquely combines dual functionality (quenching and separation), rapid target isolation (by magnetic rack, <1 min) and minimal matrix interference (validated in human sweat). The practical utility of this technology was further demonstrated through successful lactate detection in human sweat, underscoring its potential for non-invasive physiological monitoring. In sports medicine, this sensor could revolutionize training optimization by enabling real-time tracking of lactate levels, thereby preventing overtraining and enhancing athletic performance. Clinically, it offers promise for managing metabolic disorders such as lactic acidosis and monitoring chronic conditions like sepsis.

Future efforts will focus on enhancing the sensor’s sensitivity through material optimization and miniaturizing the system for portable applications. The main technical difficulty in developing this biosensor into a portable prototype lies in how to integrate nanomaterials and how to conveniently detect the changes in fluorescence signals. We can enhance the on-site testing capability of the nanoprobes by pre-encapsulating them and designing them as detachable chip card slots or test strips. For instance, APT-CS-UCNPs and Fe_3_O_4_-MoS_2_ are freeze-dried into microspheres, and protective agents are added to enhance stability. At the same time, the method of collecting sweat was upgraded to use microfluidic chips to facilitate integration with nanomaterials. These advancements aim to facilitate continuous lactate monitoring in real-world scenarios, bridging the gap between laboratory research and practical health management.

## Data Availability

The original contributions presented in the study are included in the article/supplementary material, further inquiries can be directed to the corresponding authors.
